# Deep semi-supervised learning for brain tumor classification

**DOI:** 10.1186/s12880-020-00485-0

**Published:** 2020-07-29

**Authors:** Chenjie Ge, Irene Yu-Hua Gu, Asgeir Store Jakola, Jie Yang

**Affiliations:** 1 Dept. of Electrical Engineering, Chalmers Univ. of Technoloogy, Gothenburg, 41296 Sweden; 2 Sahlgrenska University Hospital and Inst. of Neuroscience and Physiology, Sahlgrenska Academy, Gothenburg, 41345 Sweden; 3 Inst. of Image Processing and Pattern Recognition, Shanghai Jiao Tong Univ., Shanghai, 200240 China

**Keywords:** Glioma, Molecular-based brain tumor classification, Grading, MRI, Semi-supervised learning, Deep learning

## Abstract

**Background:**

This paper addresses issues of brain tumor, glioma, classification from four modalities of Magnetic Resonance Image (MRI) scans (i.e., T1 weighted MRI, T1 weighted MRI with contrast-enhanced, T2 weighted MRI and FLAIR). Currently, many available glioma datasets often contain some unlabeled brain scans, and many datasets are moderate in size.

**Methods:**

We propose to exploit deep semi-supervised learning to make full use of the unlabeled data. Deep CNN features were incorporated into a new graph-based semi-supervised learning framework for learning the labels of the unlabeled data, where a new 3D-2D consistent constraint is added to make consistent classifications for the 2D slices from the same 3D brain scan. A deep-learning classifier is then trained to classify different glioma types using both labeled and unlabeled data with estimated labels. To alleviate the overfitting caused by moderate-size datasets, synthetic MRIs generated by Generative Adversarial Networks (GANs) are added in the training of CNNs.

**Results:**

The proposed scheme has been tested on two glioma datasets, TCGA dataset for IDH-mutation prediction (molecular-based glioma subtype classification) and MICCAI dataset for glioma grading. Our results have shown good performance (with test accuracies 86.53% on TCGA dataset and 90.70% on MICCAI dataset).

**Conclusions:**

The proposed scheme is effective for glioma IDH-mutation prediction and glioma grading, and its performance is comparable to the state-of-the-art.

## Background

Gliomas are the most common brain tumors [1–3], and they make up 80% of all malignant brain tumors [4]. Symptoms depend upon their locations within the brain, and typically also vary with glioma grade and subtype. According to the World Health Organization (WHO), gliomas are graded into four classes (grades I-IV) depending on their aggressiveness. The diffuse gliomas with WHO grade II are conventionally referred to as low-grade gliomas (LGG), while high-grade gliomas (HGG) consist of those with WHO grade III and IV. Recently molecular markers have revolutionized the classification. Glioma subtype isocitrate dehydrogenase (IDH) mutations are observed in 12% of glioblastomas [5], and 70% to 80% of LGG [6]. Patients with IDH mutated gliomas survive longer than those with IDH wild-type gliomas [7–9]. Therefore, IDH mutation information plays an important role in the prognosis, diagnosis and guidance for clinical decisions. To identify glioma subtype IDH mutation, tissue diagnosis from an invasive procedure (e.g. biopsy or resection) is usually required, which might be risky to patients. Seeking effective classification methods from Magnetic Resonance Images (MRIs) may provide a non-invasive option for the identification of IDH mutation subtype. However, it is challenging as the IDH mutation information is at the molecular level. Even medical experts cannot easily observe such information from MRIs. Recently, a lot of successful machine learning methods have been proposed for predicting the glioma types such as the grade and the IDH mutation information from MRIs non-invasively, though many challenges remain that limit the performance.

## Related studies

Hand-crafted features (i.e. features designed by human experts) for machine learning techniques have been explored to characterize gliomas. Kang et al. [10] proposed to grade glioma by the histogram analysis of apparent diffusion coefficient maps. Carrillo et al. [11] proposed to predict the status of IDH mutation on gliomas by employing MRI features such as tumor size, frontal lobe localization, presence of cysts and satellite lesions. Another set of MRI features such as the pattern of growth, tumor margins, signal density and contrast enhancement were used by Qi et al. [12] on the same task of predicting IDH mutation. Yu et al. [13] explored features such as tumor location, intensity, shape, texture, and wavelet features on the classification of grade II gliomas. Zhang et al. [14] included Visually Accessible Rembrandt Images (VASARI) features for predicting IDH and TP53 mutations with SVM models. Shofty et al. [15] also extracted tumor size, location and texture features but tested 17 machine learning classifiers on 1p/19q codeletion status prediction for LGG. Zhou et al. [16] extracted histogram, shape and texture features from preoperative MRIs, and the age information is then integrated for training a random forest classifier for the prediction of IDH mutation status and 1p/19q codeletion. Although promising results have been shown in these methods, choosing which feature to use is still empirical and dataset-dependent.

Deep learning offers another way for glioma characterization by automatically learning features. Several deep learning-based glioma classification methods have been proposed in the past few years. Li et al. [17] extracted features from the last convolutional layer of a 6-layer CNN segmentation network. These features were further encoded by fisher vectors followed by feature selection and IDH mutation prediction using SVM classifiers. Chang et al. [18] proposed to apply residual CNNs to the prediction of IDH mutation using multi-institutional MRI data from four different modalities: T1 weighted, T1 weighted with contrast enhanced, T2 weighted and FLAIR (abbreviated as T1, T1ce, T2 and FLAIR in the text below). Different strategies of fusing multi-view and multimodal images were tested as well. Liang et al. [19] proposed to use 3D MRI scans with more advanced DenseNets for IDH mutation prediction. Their method was also applied to the task of glioma grading with good performance.

Although existing methods for glioma classification are promising, further improvement should be sought. Gliomas are relatively rare, and most datasets are modest in size. Since molecular markers are relatively newly implemented in routine diagnostics, many images do not have labels. The most direct way to tackle this is to only use the labeled data for training, which is not a good strategy as all the unlabeled data is wasted, given that the glioma dataset is usually not sufficiently large and thus very precious. Motivated by the medical needs, we aim to make the best use of all the images including the unlabeled ones to improve the classification performance. We propose a novel deep semi-supervised learning method for glioma classification. That is, the labels of the unlabeled data are estimated by semi-supervised learning, so that these images (with the estimated labels) can be used together with the labeled data for training a classifier. Conventional graph-based semi-supervised learning framework treats all the labeled data equally, without considering the relations between the data. For the 2D MRIs extracted from the same 3D scan of a patient, they should have the same label. To address this issue, we add the 3D-2D consistent constraint to both the graph construction and the cost function to conduct label propagation. In this way, semi-supervised learning tends to make consistent predictions on the images from the same 3D scan. Since most current glioma datasets are moderate in size, Generative Adversarial Networks (GANs) are also employed to augment more synthetic MRIs to alleviate the overfitting problem in CNNs. The main contributions of the paper include:

- Propose to use deep semi-supervised learning for estimating the labels of the unlabeled data, in order to improve the performance of glioma classification by exploring both the labeled and unlabeled data.
- Propose a 3D-2D consistent graph-based method for semi-supervised learning, by adding constraints to both the graph construction and the cost function of label propagation, so that the consistent predictions on the images from the same 3D scan can be made.
- Analyze and evaluate the performance of the proposed method by extensive empirical tests on two glioma dataset, including comparisons with some state-of-the-art methods.

## Methods

### Overview of the proposed scheme

The main idea behind the proposed scheme is to improve the performance of glioma classification by using the unlabeled data in the training dataset, whose labels are estimated by a novel graph-based deep semi-supervised learning method. The novelties include: (a) *Training dataset employs both the labeled dataset as well as the unlabeled dataset with estimated labels obtained from the proposed semi-supervised method.* By adding unlabeled data and their corresponding estimated labels to the CNN training, better performance is expected as more training data can mitigate the overfitting of deep learning. It offers more robustness and improved generalization to the CNN classifier. (b) *Labels of the unlabeled data are estimated by a graph-based semi-supervised learning method.* The 3D-2D consistent constraint is introduced to improve the convolutional graph-based label propagation framework, based on the intuition that 2D MRIs from the same 3D scan should have the same label of glioma. Such constraint is added to both the way of graph construction and the cost function of label propagation for semi-supervised learning.

**Fig. 1:**
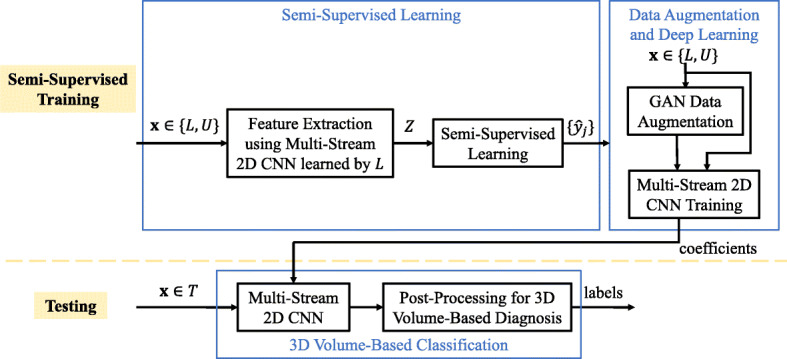
The proposed deep semi-supervised learning scheme for glioma classification, where $\mathcal {L}$, $\mathcal {U}$ and $\mathcal {T}$ denote the labeled training dataset, unlabeled training dataset and the testing dataset, $\mathcal {Z}$ denotes the feature set, and $\left \{\hat {y}_{j}\right \}$ represents the estimated labels for images in $\mathcal {U}$

The pipeline of the proposed scheme is shown in Fig. 1. It consists of three modules, semi-supervised learning, data augmentation and deep learning, and 3D volume-based classification. Multi-stream 2D CNN is first trained using only the labeled data in the training dataset. It is then used to extract features from both the labeled and unlabeled data in the training dataset. Graph-based semi-supervised learning is used to learn the estimated labels of the unlabeled data. Training data from both labeled and unlabeled sets are fed into GANs to generate synthetic MRIs for data augmentation. The labeled training dataset, unlabeled training dataset with estimated labels as well as the GAN-augmented data are used as input to multi-stream 2D CNN for learning the characteristics of gliomas. After that in the testing phase, MRI slices from the testing dataset are tested using the trained CNN, followed by post-processing to output the glioma type for each 3D brain scan. The main contributions of this paper include the graph-based semi-supervised learning and the design of the whole scheme using unlabeled training data for glioma type classification. In the following, a detailed description of the graph-based semi-supervised learning will be given.

### Graph-Based semi-supervised learning

To estimate the labels for the unlabeled data, a new graph-based semi-supervised learning method is proposed for glioma type classification. This subsection describes the graph-based semi-supervised learning method in the following three parts: problem formulation, graph construction and graph-based label propagation.

#### Problem formulation

Let $ \mathcal {X}=\left \{ \mathcal {L},~ \mathcal {U} \right \}$ be the set of all the images. The images ${\textbf x}_{i} \in \mathcal {L}$, *i*=1,⋯,*l*, are labeled as *y*_1_,⋯,*y*_*l*_ where *y*_*i*_∈{1,⋯,*c*}, and *c* is the total number of classes. The remaining images ${\textbf x}_{i} \in \mathcal {U} $, *i*=*l*+1,⋯,*n*, are unlabeled. Before semi-supervised learning, original images are usually mapped to a feature space $ \mathcal {Z}=\{ {\textbf z}_{1}, \cdots, {\textbf z}_{n} \}$ where **z**_*i*_=*f*_*θ*_(**x**_*i*_) and *f*_*θ*_(·) is the feature extraction function. In this case, we use initially trained multi-stream 2D CNN [20] for feature extraction (as shown in the deep feature extraction block in Fig. 1). The aim of the semi-supervised learning is to predict the labels $\hat {y}_{l+1}, \cdots, \hat {y}_{n}$ of the unlabeled images in $\mathcal {U}$ using the feature set $\mathcal {Z}$ from all images and the labels *y*_1_,⋯,*y*_*l*_ from the labeled images in $\mathcal {L}$.

#### Graph construction

To conduct graph-based semi-supervised learning, a graph $\mathcal {G}=(\mathcal {V},\mathcal {E})$ is defined where vertices $\mathcal {V}$ denotes the set of images and $\mathcal {E}$ is a set of graph edges. To form the edges in $\mathcal {E}$, a *k* nearest neighbour strategy is adopted by connecting each image to its *k* nearest neighbours in the feature space $\mathcal {Z}$ using the Euclidean distance. Observing that images (corresponding to 2D slices of MRIs) belonging to the same 3D scan should have the same label of glioma type, these images are connected to each other in the graph. Noting that some images from the same 3D scan are not neighbours in the feature space due to variations and differences in angles. Such an edge construction approach makes it easier for the label information to propagate among these images, so that they have a high probability to obtain the same (3D-2D consistent) label after the graph-based semi-supervised learning. The affinity matrix ${\textbf A}\in \mathbb {R}^{n \times n}$ is calculated component-wise using the Gaussian similarity in the feature space by

1 $$ a_{i,j}=\text{exp}\left(-\frac{\|{\mathbf z_{i}}-{\mathbf z_{j}}\|_{2}^{2}}{2\sigma^{2}}\right),  $$

if *i*≠*j* and **x**_*i*_,**x**_*j*_ are connected in the graph, where *σ* is the standard deviation of the Gaussian function. The affinity matrix is then converted to a symmetric version **W**=(**A**+**A**^*T*^)/2 representing the pairwise similarity between **z**_*i*_ and **z**_*j*_. The degree matrix is defined as **D**=diag(**W1**_*n*_) whose diagonal value *D*_*ii*_ is the sum of the *i*th row/column vector in **W**, and **1**_*n*_ is a *n*-dimensional vector with all-one values.

#### Graph-Based label propagation

To estimate the labels of the unlabeled images, a new graph-based label propagation method is proposed for semi-supervised learning. The idea is to propagate the label information from labeled images to unlabeled ones through a graph, with an added 3D-2D consistent constraint to further improve its performance. The cost function of the proposed method is described as

2 $$ \begin{aligned} E({\mathbf S})=&\sum_{i,j=1}^{n}{\mathbf W}_{i,j}\|\frac{{\mathbf s}_{i}}{\sqrt{D_{ii}}}-\frac{{\mathbf s}_{j}}{\sqrt{D_{jj}}}\|^{2}+\mu\|{\mathbf S}-{\mathbf Y}\|_{F}^{2}\\ &+\lambda\|{\mathbf S}-{\mathbf B}{\mathbf S}\|_{F}^{2}, \end{aligned}  $$

where **S** is the estimated labels for all images after label propagation, ${\textbf s}_{i}\in \mathbb {R}^{1\times c}$ is the one-hot vector from the *i*-th row of **S** denoting the label for the *i*-th image, *μ*>0 and *λ*>0 are the balancing weights. The one-hot label matrix **Y**_*n*×*c*_ is defined as

3 $$ {\mathbf Y}_{i,j}=\left\{ \begin{array}{ll} 1, & \text{if}~~ {\textbf x}_{i}\in \mathcal{L} ~~\text{and}~~ y_{i}=j, \\ 0, & \text{otherwise}. \end{array} \right.  $$

The first term in (2) is the smoothness constraint where images that are close to each other in the feature space have similar labels. The second term is the fitting constraint to force the labeled images to remain their labels. These two terms are adopted from [21] as the framework for label propagation. The third term is to enforce the images in a 3D scan to share the same label, and ${\textbf B}\in \mathbb {R}^{n\times n}$ is defined as

4 $$ {\mathbf B}=\left[ \begin{array}{llll} \frac1{n_{s}}{\mathbf 1_{n_{s}\times n_{s}}} & 0 &... & 0 \\ 0 & \frac1{n_{s}}{\mathbf 1_{n_{s}\times n_{s}}} &... & 0 \\... &... &... & 0\\ 0 & 0 & 0 & \frac1{n_{s}}{\mathbf 1_{n_{s}\times n_{s}}} \end{array}\right].  $$

We assume that each patient’s 3D scan contains *n*_*s*_ 2D MR image slices, and the first set of *n*_*s*_ images ${\textbf x}_{1},{\textbf x}_{2},...,{\textbf x}_{n_{s}}\phantom {\dot {i}\!}$ are from the first patient, the second set of *n*_*s*_ images ${\textbf x}_{n_{s}+1},{\textbf x}_{n_{s}+2},...,{\textbf x}_{2\times n_{s}}$ are from the second patient, and so on. The 3D scan-consistent restraint term $\|{\textbf S}-{\textbf B}{\textbf S}\|_{F}^{2}$ may be further expanded as

5 $$ \|{\mathbf S}-{\mathbf B}{\mathbf S}\|_{F}^{2}=\sum_{i=1}^{n}\|{\mathbf s}_{i}-{\mathbf s}_{mi}\|_{2}^{2},  $$

where ${\mathbf s}_{mi}=\frac 1{n_{s}}\sum _{j=\lfloor i/n_{s}\rfloor \times n_{s}+1}^{\lfloor i/n_{s}\rfloor \times n_{s}+n_{s}}{\mathbf s}_{j}$, and **s**_*mi*_ is the mean prediction of the 2D image slices **x**_*i*_ for the same 3D scan. The constraint term in (5) is a variance penalty forcing the images from the same patient to have the same label. Here we choose the variance of the predictions as the penalty, other metrics that can reflect their consistencies such as entropy may also be adopted.

To minimize the cost function, applying partial derivative to (2) with respect to **S** and setting it to zero, $\frac {\partial E({\textbf S})}{\partial {\textbf S}}=0$, lead to

$$\left({\mathbf I}-{\mathbf D}^{-\frac12}{\mathbf W}{\mathbf D}^{-\frac12}\right){\mathbf S}+\mu({\mathbf S}-{\mathbf Y})+\lambda({\mathbf I}-{\mathbf B})^{T}({\mathbf I}-{\mathbf B}){\mathbf S}=0.$$

The estimated matrix of labels **S** is obtained as

6 $$ {\mathbf S}=\mu\left[(1+\mu){\mathbf I}-{\mathbf D}^{-\frac12}{\mathbf W}{\mathbf D}^{-\frac12}+\lambda({\mathbf I}-{\mathbf B})^{T}({\mathbf I}-{\mathbf B})\right]^{-1}{\mathbf Y},  $$

where **I** is the identical matrix with dimension *n*×*n*. The symmetric normalized Laplacian matrix ${\textbf I}-{\textbf D}^{-\frac 12}{\textbf W}{\textbf D}^{-\frac 12}$ is semi-positive definite, and (**I**−**B**)^*T*^(**I**−**B**) is semi-positive definite. With *μ*>0 and *λ*>0, $(1+\mu){\textbf I}-{\textbf D}^{-\frac 12}{\textbf W}{\textbf D}^{-\frac 12}+\lambda ({\textbf I}-{\textbf B})^{T}({\textbf I}-{\textbf B})$ is positive definite and thus invertible. It is worth noting that if the number of images *n* is large, the closed-form solution in (6) is not practical. One can resort to the conjugate gradient method [22] or the iterative method [21] for an approximate solution.

The estimation of the label for the unlabeled image **x**_*i*_ is obtained from examining the label vector **s**_*i*_ such that

7 $$ \hat{y_{i}}=\mathop{\arg\max}_{j}{\mathbf s}_{i,j},~~~i\in\left\{l+1,\cdots,n\right\},  $$

where **s**_*i*,*j*_ is the *j*th element of vector **s**_*i*_.

The algorithm of the graph-based semi-supervised learning for glioma classification is summarized in Algorithm 1 below.


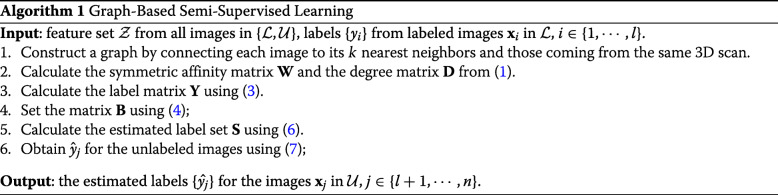


### Implementation issues

#### Multi-Stream 2D convolutional neural network (CNN) for supervised learning

**Fig. 2:**
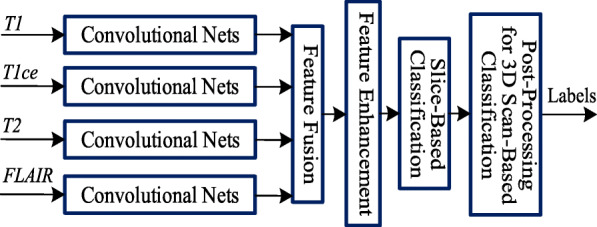
The baseline method of multi-stream 2D CNN for supervised learning and classification of gliomas in [20]

We adopt the multi-stream 2D CNN in [20] as the baseline method for the feature learning/extraction and classification of 2D MR image slices, followed by post-processing for 3D tumor type estimation, where the block diagram is depicted in Fig. 2. This baseline method contains four separate streams for learning glioma features in each modality of MR image slices followed by the fusion of four modality features. The 2D CNN of each stream has seven convolutional layers with filter size 3 ×3 in each layer. Feature map of each stream is extracted from the last convolutional layer, and then the four streams of features are fed to the feature fusion and enhancement layers. We apply a weighted sum on these features through the attention weights, which are learned adaptively according to their modality-specific characteristics. Feature enhancement layer is employed to map the fused features to a high-dimensional feature space, leading to the feature representation with complementary information from different modalities. After that, the enhanced feature map is fed to the classifier consisting of 3 fully-connected (FC) layers for the slice-based classification. Post-processing is finally conducted using majority voting on slice-based classification results, and it results in the brain tumor classification based on 3D scans.

#### Pairwise GAN for augmenting brain MRI slices

The pairwise GAN [20] is used to augment synthetic MRIs across different modalities for fake patients. It offers more robustness as GAN-augmented MRIs covers more tumor statistics according to their distributions. In pairwise GANs, two streams of GANs (generators *G*_*m*_, *G*_*n*_ and discriminators *D*_*m*_, *D*_*n*_) are interconnected. The loss function consists of adversarial loss *L*_*m*_, *L*_*n*_ and pixel-level loss *L*_1_(*G*_*m*_,*G*_*n*_), where tumor masks are used in *L*_1_(*G*_*m*_,*G*_*n*_) to enhance the pixels in the tumor area for generating more realistic tumors. For more details please refer to [20]. The GAN-augmented images are used initially for pre-training multi-stream 2D CNN for glioma feature learning and classification, and then the real MRIs are used for refined-training.

#### Estimating the labels of GAN-augmented MRI slices

In the proposed scheme, semi-supervised learning is used for estimating the labels of the unlabeled MRI slices in the training dataset, to include more data in the training dataset. Meanwhile, to check whether GAN-augmented MRI slices have the right labels, these slices are also treated as the unlabeled data in the framework of semi-supervised learning. The estimated labels of the GAN-augmented MRI slices by semi-supervised learning are further compared with their original labels to remove outliers. Only GAN-augmented slices whose original labels are the same as the estimated labels by semi-supervised learning are added to the training dataset.

## Results

### Setup, datasets and metrics

*Setup:* KERAS library [23] with TensorFlow [24] backend was used for our experiments. All experiments were done on a workstation with Intel-i7 3.40GHz CPU, 48G RAM and an NVIDIA Titan Xp 12GB GPU. Hyperparameter settings were as follows: for TCGA dataset, pretraining was applied to GAN augmented images using 100 epochs with the learning rate 1e-4 for epochs ∈[1,30], 1e-5 for epochs ∈[31,60], and 1e-6 for epochs ∈[61,100]. Refined training was then applied to the original images using 50 epochs with the learning rate 1e-5. For MICCAI dataset, pretraining was applied to GAN augmented images using 70 epochs with the learning rate 1e-4 for epochs ∈[1,50], 1e-5 for epochs ∈[51,70]. Refined training was then applied to the real images by using 70 epochs with the learning rate 1e-4 for epochs ∈[1,40], and 1e-6 for epochs ∈[41,70]. Optimizer was Adagrad. Batch size was 9. L2 regularization term was applied with parameter 1e-4. Dropout rate was set to 0.5 in the FC layers. Simple augmentation strategies such as flipping (horizontal) and shifting (max 10% of image width and height) were also used. They were realized by Keras function *ImageDataGenerator*, and only performed on the training dataset in real time to minimize the memory usage.

*Datasets:* Two datasets were used in our experiments. TCGA dataset contains 3D brain scans (i.e., 3D volume images) from TCGA-GBM [25] and TCGA-LGG [26] with IDH genotype labels. MICCAI dataset contains 3D brain scans of low-grade glioma (LGG) and high-grade glioma (HGG), downloaded from MICCAI BraTS 2017 competition [27, 28]. Both datasets contain four types of 3D brain MRI scans (T1, T1ce, T2, FLAIR) and tumor segmentation results. Although TCGA and MICCAI datasets have some overlap on IDH genotype, the class labels for MICCAI dataset used in our study are only related to low and high grades of gliomas (LGG/HGG), not genotypes.For TCGA dataset the aim is to classify/predict the tumor subtypes in the molecular levels by using multimodal MRIs. For MICCAI dataset the aim is to classify the glioma into low and high grades. Detailed information of two datasets is given in Table 1.

**Table 1:** Information of the two datasets based on 3D brain scan, where 9 image slices per 3D scan were used in all our experiments

Dataset	Tumor	#Patients	#3D scans	#3D scans	#3D scans	#3D scans
	type		(T1/T1ce/	for training	for validation	for testing
			T2/FLAIR)	(origial/GAN	(original)	(original)
				augmented)		
TCGA	IDH mutation	55	55	33/99	6	16
	IDH wild-type	112	112	66/198	13	33
MICCAI	HGG	210	210	126/126	21	63
	LGG	75	75	45/45	7	23

Further, the training/validation/testing sets were patient-wise partitioned according to 60%,10%,30% approximately, and 2/3 of the original scans in the training datasets were labeled, and the remaining 1/3 were set as unlabeled

**Fig. 3:**
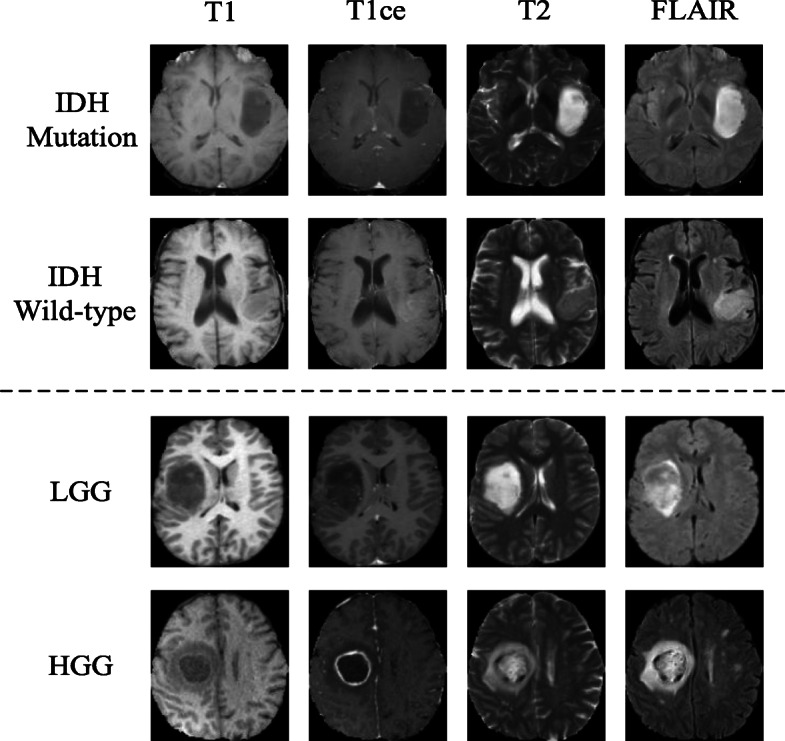
Examples of 2D MRI slices from IDH mutation/wild-type gliomas and LGG/HGG in four modalities

Example images of four modalities in two classes are shown in Fig. 3. Since the volume of tumor is usually small/medium in size, 9 slices that contain gliomas were extracted from each individual scan. This was done for both classes. For focusing feature learning on the tumor areas instead of the whole brain, tumor masks were applied to enhance the tumor feature learning by multiplying the background pixels by a factor of 1/3. For all our experiments, dataset was partitioned into 3 subsets: training (60%), validation (10%) and testing (30%). All 2D image slices in these 3 subsets were partitioned *according to patients*, i.e., images from the same patient were kept together in either training subset or the testing subset, as such partition was clinically important. 2/3 of the training dataset (for both original and GAN augmented data) was labeled and the other 1/3 was set as unlabeled.

*Metrics for performance evaluation:* Cross-entropy loss was used as the loss function for evaluating the performance of CNN training. To evaluate the performance of the glioma classification, we used the accuracy, sensitivity and specificity in (8) as the criteria on the results obtained from the test set averaged over 5 runs,

8 $$ \begin{array}{ll} \text{Accuracy}=\frac{\textrm{TP+TN}}{\textrm{TP+FP+TN+FN}}, \\ & \\ \text{Sensitivity}=\frac{\text{TP}}{\textrm{TP+FN}},&\text{Specificity}=\frac{\text{TN}}{\textrm{FP+TN}}, \\ \end{array}  $$

where one has the following definitions by selecting IDH mutation/LGG as the target class:*True positive (TP)*: IDH mutation glioma/LGG was correctly classified as IDH mutation/LGG.*False positive (FP)*: IDH wild-type glioma/HGG was incorrectly classified as IDH mutation/LGG.*True negative (TN)*: IDH wild-type glioma/HGG was correctly classified as IDH wild-type/HGG.*False negative (FN)*: IDH mutation glioma/LGG was incorrectly classified as IDH wild-type/HGG.

### Performance of the proposed method

**Fig. 4:**
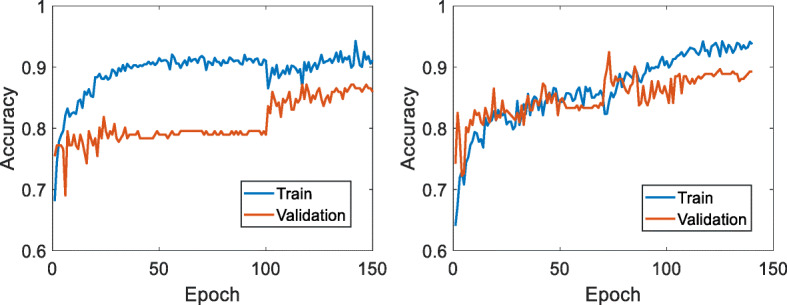
Training and validation curves (accuracy vs. epoch) on the two datasets. Left: TCGA dataset, where epochs 1-100 were from the pre-training and epochs 101-150 were from the refined training; Right: MICCAI dataset, where epochs 1-70 were from the pre-training and epochs 71-140 were from the refined training

To test the effectiveness of the proposed deep semi-supervised learning for classifying gliomas, experiments were conducted with 5 runs on two datasets, where the partitions of training, validation and testing subsets were done randomly in each run. Figure 4 shows the training and validation curves in the training process for two datasets from the first run.

Observing Fig. 4, training has converged for both datasets, with high validation accuracies showing good generalization ability for the unseen data. The gap between the training and validation performance also indicates small overfitting probably due to the moderate size of training dataset.

Table 2 shows the test results and performance obtained from the proposed scheme on TCGA and MICAII datasets. Table 2 (a) shows the performance of the 5 runs on testing sets with the mean accuracy as well as the standard deviation, (b) and (c) show the confusion matrices from test results on two datasets.

**Table 2:** Test results and performance obtained from the proposed scheme on TCGA and MICAII datasets

(a)Average accuracy, sensitivity and specificity on the test sets, where			
the standard deviation is included in (·) after each performance value.			
Dataset	Accuracy (|*σ*|)	Sensitivity (|*σ*|)	Specificity (|*σ*|)
TCGA	86.53(4.24)	73.75(8.15)	92.73(3.45)
MICCAI	90.70(1.42)	84.35(6.59)	93.01(1.42)
(b) The confusion matrix from test results on the TCGA dataset.			
True ∖ Classified	IDH mutation	IDH wild-type	
IDH mutation	73.75	26.25	
IDH wild-type	7.27	92.73	
(c) The confusion matrix from test results on the MICCAI dataset.			
True ∖Classified	HGG	LGG	
HGG	93.01	6.99	
LGG	15.65	84.35	

All results were obtained by averaging over 5 runs, and |*σ*| is the standard deviation, all values in the tables (a) (b) and (c) are in percentage %

Observing Table 2(a), the proposed method was shown to be effective according to the test accuracies on two datasets. For TCGA dataset, a relatively high average accuracy of 86.53% was achieved, with average sensitivity 73.75% and specificity 92.73%. For MICCAI dataset, average test accuracy was 90.70% with sensitivity 84.35% and specificity 93.01%. Test results from both datasets were reasonably well. Since the tests on TCGA dataset were aimed at predicting of IDH genotypes, which was a more challenging task than that of tumor grading on MICCAI dataset, the slightly lower test performance on TCGA dataset than that on MICCAI dataset (86.53% vs. 90.70%) was expected. Further, observing the confusion matrix in Table 2(b), relatively higher accuracy was on IDH wild-type class but lower accuracy on IDH mutation class, indicating more false alarm on IDH wild-type class. From Table 2(c) one can see the accuracy on HGG was higher than that of LGG. The unbalanced test accuracies on the two classes, and the difference between specificity and sensitivity from two datasets were likely caused by the unbalanced number of brain MRIs in the two classes (both IDH mutation/wild-type and HGG/LGG) as shown in Table 1.

### Comparison with baseline methods

We define three different methods for evaluating the performance of the proposed semi-supervised learning scheme for glioma classification:*Baseline-1 method*: the training dataset only consisted of the original labeled 2D image slices $\mathcal {L}$ plus GAN augmented images based on $\mathcal {L}$, where pretraining was applied on GAN-augmented image set. *Proposed scheme*: the training dataset consisted of the original labeled 2D image slices $\mathcal {L}$ and unlabeled ones $\mathcal {U}$ whose labels were estimated from graph-based semi-supervised learning. Pretraining was applied on the GAN-augmented images where a small number of outlier images from GANs were removed.*Baseline-2 method*: the training dataset consisted of the original labeled 2D image slices $\mathcal {L}$ and $\mathcal {U}$ in which the ground-truth labels were used. Pretraining was applied on the GAN-augmented image slices where a small number of outlier images of GANs were removed.

**Fig. 5:**
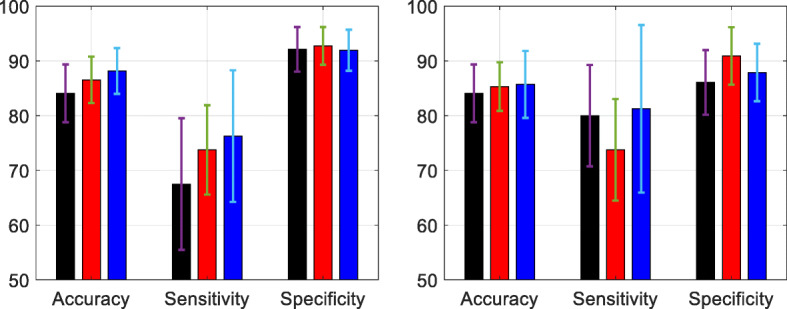
Performance on the TCGA dataset, where the results were obtained from the test set averaged over 5 runs. Left: performance where GAN augmented data was added in the training set; Right: performance where no GAN augmented data was used in the training set. Red: from the proposed semi-supervised scheme; Black: from baseline-1 method; Blue: from baseline-2 method

Results on two different settings, with and without adding GAN-augmented data for the pre-training, are shown in Fig. 5, where the average results over 5 runs as well as the standard deviation |*σ*| for the overall accuracy, sensitivity, and specificity are included.

Observing Fig. 5, the proposed scheme on two cases (with and without GAN) achieved better performance than the baseline-1 method according to the accuracy, while slightly lower performance than the baseline-2 method. This result indicates that the proposed semi-supervised learning scheme is effective in estimating the labels for the unlabeled training data $\mathcal {U}$, which has contributed to the performance improvement as compared with the baseline-1 method without using unlabeled data in $\mathcal {U}$. The small gap between the proposed method and baseline-2 method also suggests that the estimated labels by semi-supervised learning were close to those of the real labels, thus leading to the similar performance on the testing sets. One can see from the right image in Fig. 5 that sensitivity has dropped compared to the two baseline methods, with increased specificity. This was probably due to the similar reason (as in Table 2) of using imbalanced number of training data between the two classes.

**Fig. 6:**
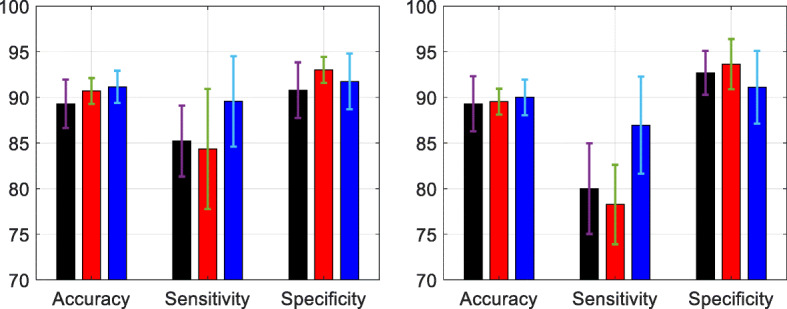
Performance on the MICCAI dataset, where the results were obtained from the test set averaged over 5 runs. Left: performance where GAN augmented data was added in the training set; Right: performance where no GAN augmented data was used in the training set. Red: from the proposed semi-supervised scheme; Black: from baseline-1 method; Blue: from baseline-2 method

A similar comparison between the proposed scheme and the two baseline methods was also done by using the test results averaged over 5 runs on the MICCAI dataset, as shown in Fig. 6.

Observing Fig. 6, the proposed scheme has achieved improved accuracy compared to baseline-1 method but lower accuracy than baseline-2 method for both cases. The sensitivity rate has dropped in the proposed method with additional gain in the specificity rate. One can observe that both in Figs. 5 and 6, there was a relatively big difference between the sensitivity and specificity values. This was probably due to the imbalanced training data in the two classes (for both TCGA and MICCAI dataset). Further, the semi-supervised learning has generated increased performance for the class with more training data, at the cost of decreased performance for the class with less training data.

### Impact of adding GAN-augmented images in the training

**Fig. 7:**
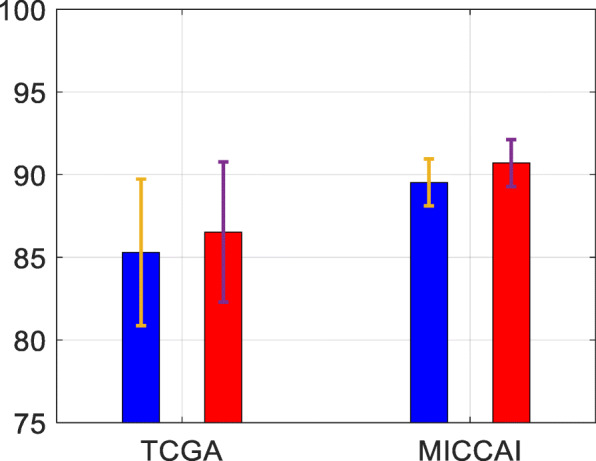
Test accuracy from the proposed scheme on TCGA and MICCAI datasets, with/without adding GAN augmented images in the training set. Blue: without using GAN augmented images; Red: with GAN augmented images

To evaluate the impact of adding GAN-augmented MR image slices in the training dataset, a comparison was made for the methods with and without including GAN-augmented images in the training datasets of TCGA and MICCAI. Mean accuracy and standard deviation averaged over 5 runs are shown in Fig. 7 and Table 3.

**Table 3:** Test performance from the proposed scheme on TCGA and MICCAI datasets with and without adding GAN-augmented images in the training

Dataset	GAN	Accuracy(|*σ*|)	Sensitivity(|*σ*|)	Specificity(|*σ*|)
TCGA	Without	85.30(4.43)	73.75(9.27)	90.91(5.25)
	With	**86.53**(4.24)	73.75(8.15)	**92.73**(3.45)
MICCAI	Without	89.53(1.42)	78.26(4.35)	**93.65**(2.75)
	With	**90.70**(1.42)	**84.35**(6.59)	93.01(1.42)

Results are shown in mean value (standard deviations |*σ*|) over 5 runs, all values are in percentage %. The best results in accuracy/sensitivity/specificity are marked in boldface

Observing Fig. 7, adding GAN-augmented data in the pre-training before refined training on real brain MRI data has improved test accuracies on both datasets. Observing the test results on TCGA dataset in Table 3, the proposed method by adding GAN-augmented training data has led to improved average accuracy and an improved specificity rate. From the test results of MICCAI dataset in Table 3, the proposed method by adding GAN-augmented data has resulted in the improved sensitivity by 6.09% and a slight drop in specificity. Overall, adding GAN-augmented data in the training dataset has shown to be effective for improving the test performance of glioma classification on these two datasets.

### Comparison to the state-of-the-art methods

The performance of several existing methods on two datasets for classifying glioma types with IDH mutation/wild-type and glioma grades are shown in Tables 4 and 5. All the other methods in Tables 4 and 5, used for comparison with the proposed method, have applied supervised training by using fully annotated datasets. It is worth noting that results from [13, 14, 17, 18] in Table 4, and the results from [29] in Table 5 can only be used as an indication since they were applied in different datasets. [30] used a very different approach as our proposed scheme here, which applied a 3D multiscale CNN network directly on 3D T1ce MR images, with fully annotated training dataset.

**Table 4:** Comparison of IDH mutation/wide type classification from the proposed scheme and some existing methods

Method	# Patients with IDH	Test
	mutation/wild-type	Accuracy (%)
Liang [19]	55/112	84.60
**Proposed**	55/112	**86.53**
Yu [13]	76/34	80.00
Zhang [14]	70/33	80.00
Li [17]	89/30	86.55
Chang [18]	233/263	85.70

Noting that [13, 14, 17, 18] were applied to different datasets, hence they were only used here as the indication/reference to the state-of-the-art in IDH mutation/wild-type classification. The performance of the proposed method is in boldface

**Table 5:** Comparison of low/high grade glioma classification from the proposed scheme and some existing methods

Method	# Patients with	Test
	HGG/LGG	Accuracy (%)
Pan [29]	188/25	73.33
Ge [30]	210/75	89.47
**Proposed**	210/75	**90.70**

The performance of the proposed method is in boldface

Observing Tables 4 and 5, it is shown that the proposed method is better than those in [19, 30] in terms of test accuracy. It is also indicated that the proposed method has reached high performance as comparing with the methods in [13, 14, 17, 18], noting the results were obtained from different datasets with different number of patients. These comparisons have also indicated that the proposed deep semi-supervised learning is effective in estimating the labels of the unlabeled data, with the performance reaching the existing state-of-the-art methods.

## Discussion

From our experimental results, some insights can be gained from the proposed scheme:

- High overall performance was achieved on two datasets for two different glioma classification tasks: molecular-based glioma subtype classification (to classify IDH mutation/wild-type), and glioma grading (to classify high-grade/low-grade gliomas).
- Semi-supervised learning scheme is effective for estimating the labels for the unlabeled dataset. With the unlabeled data and the estimated labels obtained from semi-supervised learning, the proposed scheme achieved improved performance compared to the baseline without using any unlabeled data. This indicates that the proposed semi-supervised glioma classification scheme is useful in real scenarios when part of the labels in the dataset is missing and the labeled dataset is small.
- Adding GAN-augmented data in the training dataset for pretraining has improved the classification performance on the testing set. It suggests that GANs are useful in augmenting synthetic MRIs, and pretraining with GAN augmented data followed by refined training with real MRI data, has improved the generalization performance on the unseen testing dataset for glioma classification.
- Large imbalanced training data between classes is undesirable, as this may result in relatively large differences of performance between individual classes.
- Comparison of performance with several state-of-the-art methods has indicated that the proposed semi-supervised approach has reached comparable performance to those of fully supervised ones.

Limitation: The imbalance of training data (including GAN generated training data) in two different classes has caused one class with relatively lower performance, consequently, it has affected the average test performance. Possible solutions could be to extract more 2D MRI slices for the class with smaller number of patients, and to explore other types of GANs allowing augmenting more MRI slices covering a wider range of tumor statistics.

## Conclusion

The proposed scheme has been tested on two glioma classification datasets, and results have shown the effectiveness of the proposed scheme with high average test accuracies (86.53% for two molecular-based subtypes IDH mutation/wild-type, and 90.7% for high-grade/low-grade gliomas). Using graph-based semi-supervised learning for estimating the labels of the unlabeled data in the training dataset has resulted in the increased performance on the testing dataset. This indicates that the proposed scheme is useful in the real scenarios when some labels of the data in a dataset are missing. Adding GAN-augmented data in the training dataset is useful for increasing the generalization performance on the testing dataset. Finally, comparisons with several different methods, although based on different datasets, have shown that the proposed method is comparable to the state-of-the-art. Limitation of the method is also discussed. Future work will be on extending glioma subtypes to include both IDH genotype and 1p/19q codeletion status, and incorporating patient side information (e.g. ages, survival years).

## Abbreviations

MRI: Magnetic Resonance Image
T1: T1 weighted MRI
T1ce: T1 weighted MRI with contrast enhanced
T2: T2 weighted MRI
FLAIR: Fluid-Attenuated Inversion Recovery
LGG: Low-grade glioma
HGG: High-grade glioma
IDH: Isocitrate dehydrogenase
GAN: Generative Adversarial Network
CNN: Convolutional Neural Network
